# Efficacy and safety of iguratimod plus corticosteroid as bridge therapy in treating mild IgG4‐related diseases: A prospective clinical trial

**DOI:** 10.1111/1756-185X.13633

**Published:** 2019-06-27

**Authors:** Panpan Zhang, Yiyi Gong, Zheng Liu, Yanying Liu, Wei Lin, Jieqiong Li, Mu Wang, Xiaowei Liu, Yunyun Fei, Hua Chen, Linyi Peng, Jing Li, Jiaxin Zhou, Qun Shi, Xuan Zhang, Min Shen, Xiaofeng Zeng, Fengchun Zhang, Yongzhe Li, Yan Zhao, Wen Zhang

**Affiliations:** ^1^ Department of Rheumatology Peking Union Medical College Hospital Chinese Academy of Medical Sciences & Peking Union Medical College Beijing China; ^2^ Key Laboratory of Rheumatology and Clinical Immunology Ministry of Education Beijing China; ^3^ Central Research Laboratory Peking Union Medical College Hospital Chinese Academy of Medical Sciences & Peking Union Medical College Beijing China; ^4^ Department of Rheumatology Tianjin Medical University General Hospital Tianjin China; ^5^ Department of Rheumatology Peking University People's Hospital Beijing China; ^6^ Department of Rheumatology Hebei General Hospital Hebei China; ^7^ Department of Stomatology Peking Union Medical College Hospital Chinese Academy of Medical Sciences & Peking Union Medical College Beijing China; ^8^ Department of ophthalmology Peking Union Medical College Hospital Chinese Academy of Medical Sciences & Peking Union Medical College Beijing China; ^9^ Department of Clinical Laboratory Peking Union Medical College Hospital Chinese Academy of Medical Sciences & Peking Union Medical College Beijing China

**Keywords:** B lymphocytes, IgG4‐RD, iguratimod, treatment

## Abstract

**Aim:**

The purpose of this study is to evaluate the therapeutic efficacy and safety of iguratimod plus corticosteroid as bridge therapy in the treatment of mild immunoglobulin G4‐related disease (IgG4‐RD).

**Methods:**

Newly diagnosed IgG4‐RD patients, without internal organ involvement were enrolled. Patients were given one dose of diprospan, intramuscular injection, and iguratimod, 25 mg, twice daily, for 24 weeks and were followed up at 0, 12 and 24 weeks. Follow‐up indexes included IgG4‐RD responder index (IgG4‐RD RI), serology and imaging, plasma cytokines and adverse drug effect. Flow cytometry was performed for T, B cell subsets and plasma was collected for liquid chromatography mass spectrometry (LC‐MS)‐based metabolomic profiling and data processing.

**Results:**

Thirty patients were enrolled. At week 24, 9 (30.0%) patients achieved complete response, 17 (56.7%) patients with partial response, and 4 (13.3%) patients had no response to treatment. IgG4‐RD RI, serum IgG and IgG4 levels decreased significantly at weeks 12 and 24 after treatment, as well as CD3+ CD8+ T cells, plasmablast/plasma cells and memory B cells. The LC‐MS based plasma metabolomic profiles revealed significant changes between untreated patients and healthy donors, which became much similar to normal states after treatment.

**Conclusion:**

Iguratimod plus corticosteroid as bridge therapy is effective for the treatment of mild IgG4‐RD, it can improve the clinical symptoms, reduce serum IgG and IgG4 levels, especially plasmablasts/plasma cells and memory B cells. In addition, the metabolite profiling became similar to normal controls after treatment.

## 1 INTRODUCTION

Immunoglobulin G4‐related disease (IgG4‐RD) is a newly defined chronic fibro‐inflammatory disorder characterized by elevated serum IgG4 levels, tumefactive lesions with a dense lymphoplasmacytic infiltration rich in IgG4 positive plasma cells and storiform fibrosis of related organs.^1^, ^2^, ^3^ Glucocorticoids are the first‐line agents for the treatment of IgG4‐RD;^4^, ^5^, ^6^, ^7^ however, in order to maintain long‐term disease stability and avoid disease relapse, usually glucocorticoids maintenance therapy should last for a long period, which may induce various glucocorticoid‐associated adverse reactions. For some IgG4‐RD patients with mild symptoms, such as swelling of the lacrimal glands, submandibular glands, parotid glands and nasal sinus, without internal organ damage, long‐term glucocorticoids therapy for mild symptoms may have a low benefit/risk ratio. Further, a substantial proportion of patients cannot tolerate glucocorticoids.

Iguratimod (T‐614) is a new small molecule compound with anti‐inflammation and immune regulation functions which have a definite disease‐modifying effect in both animal rheumatoid arthritis (RA) models and patients.^8^, ^9^, ^10^, ^11^ In vitro studies had demonstrated that iguratimod has an anti‐inflammatory role by inhibiting tumor necrosis factor (TNF), interleukin (IL)‐1β, IL‐6, IL‐8, and monocyte chemotactic protein‐1 production.^10^ Furthermore, iguratimod could reduce the levels of serum IgG, IgM, and IgA in RA patients.^9^, ^10^ Iguratimod could also attenuate proteinuria and kidney injury in MRL/lpr lupus mice, and improve serum markers such as anti‐double‐stranded DNA (anti‐dsDNA) and serum C3.^12^

In order to investigate an optimum treatment regime for mild IgG4‐RD patients, we launched an investigator‐initiated study; after treatment with one dose of diprospan, patients were given iguratimod 25 mg, twice a day, orally. The aim of this study was to evaluate the therapeutic efficacy and safety of iguratimod plus corticosteroid as bridge therapy in the treatment of IgG4‐RD with mild symptoms.

## 2 METHODS

### 2.1 Study design

This study was conducted in Peking Union Medical College Hospital (PUMCH) between 2017 and 2018. The protocol was approved by the Ethics Committee of PUMCH (No. JS‐1404). The study was conducted in compliance with the Declaration of Helsinki and is registered at http://clinicaltrials.gov (NCT03368274). All enrolled patients and healthy controls consented to attend this study and provided signed written informed consent.

Newly diagnosed IgG4‐RD patients, with mild but progressive superficial glands swelling were enrolled in this study, and were treated with 1 dose of diprospan, 1 mL (containing 5 mg betamethasone dipropionate and 2 mg betamethasone sodium phosphate), and concomitantly took iguratimod 25 mg, twice a day, orally. Patients were evaluated at baseline, 12 and 24 weeks of follow‐up. Blood and urine routine tests, erythrocyte sedimentation rate (ESR), high‐sensitivity C‐reactive protein (hsCRP), serum immunoglobulin, IgG subclass and IgE were measured; organ involvement was evaluated by ultrasound scanning, computed tomography (CT), or magnetic resonance imaging, or positron emission tomography/CT (PET/CT). The treatment efficacy was evaluated by IgG4‐RD responder index (IgG4‐RD RI, 2012 version, each affected organ was individually scored)^13^ and physician global assessment (PGA, scored as 0‐10 cm). T cell subpopulations, B cell subpopulations plasma cells, as well as serum cytokines, including IL‐1β, IL‐17A, TNF‐α, transforming growth factor (TGF)‐β1, interferon (IFN)‐γ were measured at baseline and at 3 months by flow cytometry and cytometric beads array (CBA). In addition, patients’ serum metabolomics before and 3 months after treatment were analyzed.

Peripheral blood from healthy donors was collected for comparison of cytokines and T cell subpopulations.

### 2.2 Inclusion and exclusion criteria

Inclusion criteria: enrolled mild IgG4‐RD patients had to fulfill the following four requirements: (a) Mikulicz disease following the diagnostic criteria of IgG4‐RD (2011),^14^ with/without nasosinusitis or lymph nodes swelling; (b) without internal organs affected; (c) with slow but active disease progression; and (d) ages of patients were between 18 and 70 years. Exclusion criteria were as follows: (a) vital organs‐related, including autoimmune pancreatitis, retroperitoneal fibrosis, sclerosing cholangitis, lung disease, kidney‐affected and hypophysitis, and so on; (b) combined with other connective diseases; (c) with tumors; (d) pregnant or planning to be pregnant; (e) active infection, including hepatitis B virus, hepatitis C virus, and tuberculosis; (f) leukocytopenia, impairment of liver and kidney functions; and (g) allergy to iguratimod or cannot tolerate iguratimod.

Thirty untreated patients fulfilled the inclusion criteria and without exclusion criteria were enrolled in this clinical trial.

### 2.3 Primary and secondary outcomes

The primary outcome of this study was to evaluate the response rate at 24 weeks of treatment. Secondary outcome included changes of IgG4‐RD RI, PGA, relapse rate, serum IgG, IgG subclass, changes of B cell, T cell subpopulations, cytokines and metabolite profiles. Side effects were recorded as well.

### 2.4 Efficacy assessment

Treatment response was assessed by evaluating the changes of IgG4‐RD RI scores and was divided into three types: complete response (CR), partial response (PR) and no effect (NE, including no improvement or exacerbation). IgG4‐RD RI scores <3 and declining ≥2 were recognized as a CR; IgG4‐RD RI scores declining ≥2 but remaining ≥3 were recognized as a PR. If a patient's IgG4‐RD RI score was 3 points at the beginning, PR was considered as a 1 point decrease after the therapy. Patients with lack of apparent changes in mass sizes and/or clinical manifestations and IgG4‐RD RI scores declining <2 were considered to be NE.^4^, ^15^

The relapse of IgG4‐RD included two types, clinical relapse and serological relapse.^4^ Clinical relapse was defined as recurrence or aggravation of clinical symptoms or imaging findings with or without IgG4 concentrations elevation. Serological relapse was defined as clinical symptoms remained stable while serum IgG4 level increased more than 1 score in IgG4‐RD RI.

### 2.5 Safety assessment

Safety was assessed using adverse events reports, physical examinations, and laboratory tests including hematology, liver and kidney function. These safety assessments were undertaken at screening and at all the follow‐up periods.

### 2.6 CBA, enzyme‐linked immunosorbent assay (ELISA), and flowcytometry

Cytometric beads array kits of human IL‐1β, IL17A, TNF‐α, TGF‐β and IFN‐γ were from BD Biosciences. Human B cell activating factor (BAFF) ELISA kit was from Rockland, USA.

Peripheral blood mononuclear cells from IgG4‐RD patients and healthy controls were separated by Ficoll gradient centrifugation. Antibodies and staining for cell subsets are shown in Data S1. T cells were defined as CD3+ T cells, CD3+ CD4+ T cells, CD3+ CD8+ T cells, CD4+ CXCR5+ TFH (T follicular helper) cells, CD4+ CXCR5+ PD‐1+ /ICOS+ TFH cells, CD4+ IFN‐γ+ Th1 cells, CD4+ IL17A+IFNγ± Th17 cells. B cell subsets were defined as CD19+ CD24‐CD38hi plasmablast/plasma cells, CD19+ CD27hiCD38hi plasmablasts, CD19+ IgD‐CD38hi plasmablasts, CD19+ IgD+CD38± naïve B cells, CD19+ IgD‐CD38‐CD27+ memory B cells. Plasma cells were defined as CD38+ CD138+.

### 2.7 Metabolomics analysis

Serum metabolites was extracted by methanol (pre‐chilled to −80°C) to make a final 80% (v/v) methanol serum extract. Samples were stored at −20°C, dried, and resuspended in 50% (v/v) methanol solution until liquid chromatography tandem mass spectroscopy (LC‐MS/MS) analysis. A pool of serum extracts from all samples were used as the quality control for LC‐MS optimizing and normalizing.^16^

For LC‐MS/MS analysis, the separation was carried out on an ACQUITY Ultra‐performance LC (UPLC)^®^BEH C18 column (1.7 μm, 2.1 × 100 mm, Waters Corporation). Both ionization modes were performed with gradient elution using (A) water with 0.1% formic acid and (B) acetonitrile with 0.1% formic acid as the mobile phase. The total analysis time lasted 20 minutes at the flow rate of 0.4 mL/min. A XevoG2‐XS Q‐TOF Mass Spectrometer (Waters Corporation) was connected to the UPLC system. MS data were acquired from m/z 50 to m/z 2000 in the MS^E^ full scan mode with the acquisition rate at 0.2 s/scan. During MS analysis, a leucine‐enkephalin calibrant solution (200 ng/mL) was continuously infused into the MS to ensure mass accuracy.

The MS raw data were processed using Progenesis QI software (Waters Corporation) to perform peak detection and using EZinfo Ver. 3.0 software (Waters Corporation) for pattern recognition and principal component analysis. Further information and details are shown in Data S1.

### 2.8 Statistical analysis

Statistical analyses were performed using SPSS Statistics version 17.0 software (SPSS Inc.) and Prism software version 6.1 (GraphPad Software). Data of normal distribution are reported as mean ± SD. Normal distribution data between two groups were analyzed using independent‐samples *t* tests or paired samples *t* tests, and one‐way analysis of variance was used to compare among groups. Categorical data were analyzed by Chi‐square test. Non‐normal distribution data were analyzed by rank sum test. Laboratory and lymphocyte subsets of four patients who withdrew from iguratimod treatment were not included. A 2‐tailed *P* value <0.05 was considered of significance.

## 3 RESULTS

### 3.1 Patients’ clinical characteristics and demographics

#### 3.1.1 Demographics of IgG4‐RD patients

In total, 30 untreated IgG4‐RD patients were enrolled in this study. The age was 50.8 ± 10.2 years with disease duration of 31.5 (interquartile range 8‐66) months. The male/female ratio was 1:1. Eighteen (60%) patients had a history of allergy. The percentage of allergic rhinitis, asthma, drug allergy, dust mite allergy and eczema were 33.3%, 13.3%, 6.6%, 3.3% and 3.3%, respectively.

Twenty‐five (83.3%) patients finished this study. Four patients withdrew because of no response or exacerbation, one patient with good response stopped iguratimod treatment at 3 months because of economic reasons.

#### 3.1.2 Clinical characteristics of IgG4‐RD

The percentages of affected organs, including lacrimal glands, submandibular glands, lymph nodes, parotid glands and nasal/paranasal sinus, were 76.7%, 53.3%, 36.7%, 10% and 53.3%, respectively. All patients had slow but active disease progression, and needed to be treated; none of them had internal organs involvement.

### 3.2 Treatment efficacy

#### 3.2.1 Primary endpoint

After injection of diprospan and concomitantly iguratimod, all patients (including four patients who withdrew from the study) had alleviation of swelling glands and improvements of nasosinus symptoms within 1 week, 26 (86.7%) patients’ clinical symptoms remained alleviated at 3 months, 25 (83.3%) patients remained stable or further improved at 6 months. Five patients withdrew between 2 and 3 months. One patient who responded well stopped iguratimod treatment at week 12 for problems with the cost; he was then given leflunomide and kept stable until 24 weeks. Four patients relapsed or exacerbated 1 month later. In three of them, the lacrimal or salivary glands enlarged again, they were given prednisone 30 mg/d, combined with MTX 15 mg/wk, or leflunomide 20 mg/d, or iguratimod 25 mg twice a day. One patient exacerbated with new onset of interstitial lung disease; he was then given prednisone 50 mg/d and cyclophosphamide 100 mg/d. They all improved.

At week 12, 10 (33.3%) and 16 (53.3%) patients achieved CR and PR, respectively. At week 24, 9 (30.0%) remained CR, 17 (56.7%) patients got PR, 4 (13.3%) were NE.

Except for those four patients who had clinical relapse, among the remaining patients, 5 (16.7%) patients had serological relapse at 24 weeks.

#### 3.2.2 Secondary endpoint

##### Changes of laboratory examinations

**Figure 1:**
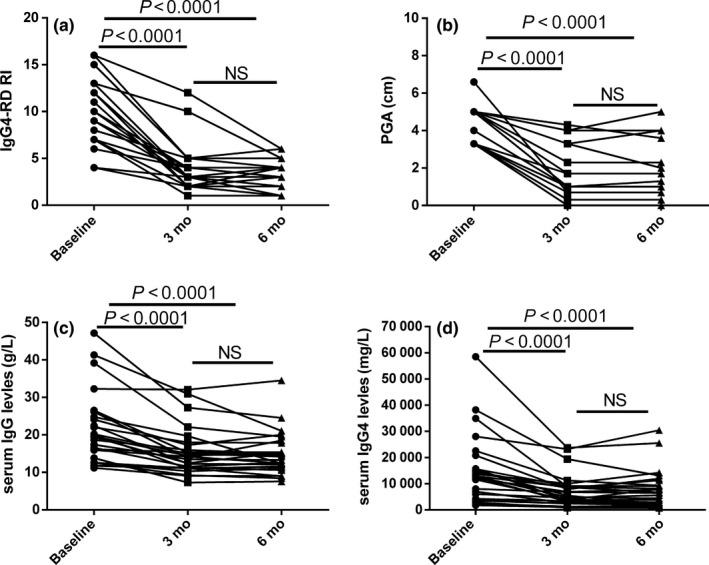
Disease activity and laboratory parameters before and after treatment. A‐D represent the changes of immunoglobulin G4‐related disease responder index (IgG4‐RD RI), physician global assessment (PGA), serum IgG and IgG4 at baseline, 3 and 6 mo of treatment, respectively

Baseline hemoglobin (HGB), white blood cells, lymphocytes and percentage of eosinophil were 138.8 ± 17.2 g/L, 6.57 ± 1.85 × 10^9^/L, 2.11 ± 0.69 × 10^9^/L and 2.4% (Q1‐Q3, 1.65%‐4.25%), respectively. ESR, hsCRP, serum IgG, IgA, IgM, IgG subclass from IgG1 to IgG4 and total IgE at baseline and after treatment are shown in Table 1. Serum IgG, IgA, IgG4, IgE and ESR were decreased significantly after treatment of 3 and 6 months (Table 1). Compared with baseline IgG (22.16 ± 9.54 g/L), serum IgG levels at 12 weeks (15.89 ± 6.88 g/L) and 24 weeks (15.69 ± 6.72 g/L) were significantly decreased, *P *=* *0.007 (Table 1, Figure 1). Meanwhile, after treatment, serum IgG4 levels decreased from 8770 (7705‐10 900) mg/L to 4725 (2738‐8748) mg/L at 12 weeks and 6020 (2613‐11 450) mg/L at 24 weeks, *P *=* *0.01 (Table 1, Figure 1). After 12 weeks treatment, 88.5% of patients’ serum IgG4 had a reduction of more than 30%, 42.3% of patients’ serum IgG4 had a reduction of more than 50%, and 11.5% of patients’ serum IgG4 returned to normal. After treatment for 24 weeks, 69.6% of patients’ serum IgG4 had a reduction of more than 30%, 43.5% of patients’ serum IgG4 had a reduction of more than 50%, and 13% of patients’ serum IgG4 returned to normal. There was no statistical significance of serum IgG and IgG4 between 12 weeks and 24 weeks of treatment.

**Table 1:** Comparison of clinical parameters at baseline, after treatment of 12 and 24 wk

Laboratory parameters	Baseline	12 wk	24 wk	*P* value
HGB (g/L)	138.8 ± 17.2	140.3 ± 15.8	139.2 ± 18.9	0.946
WBC (10^9^/L)	6.57 ± 1.85	7.53 ± 1.79	6.71 ± 1.49	0.124
Lymphocytes (10^9^/L)	2.11 ± 0.69	2.27 ± 0.73	2.27 ± 0.76	0.306
PLT (10^9^/L)	247 ± 57	245 ± 60	225 ± 52	0.352
Eosinophils %, M (Q1‐Q3)	2.4 (1.65‐2.45)	2.4 (1.70‐4.55)	1.7 (1.0‐3.4)	0.695
ESR, M (Q1‐Q3) mm/h	13 (9‐28)	7 (3‐18)	8 (5‐11)	0.045^a^
hsCRP, M (Q1‐Q3) mg/L	0.99 (0.47‐2.31)	1.05 (0.41‐1.60)	0.83 (0.45‐4.86)	0.893
IgG (g/L)	22.16 ± 9.54	15.89 ± 6.88	15.69 ± 6.72	0.007^a^
IgA (g/L)	2.10 ± 1.09	1.78 ± 0.95	1.57 ± 0.85	0.01^a^
IgM (g/L)	0.97 ± 0.66	0.74 ± 0.46	0.74 ± 0.48	0.541
IgG1, M (Q1‐Q3) mg/L	8770 (7705‐10 900)	6690 (6405‐12 585)	7155 (6502‐7530)	0.416
IgG2, M (Q1‐Q3) mg/L	5160 (4350‐7335)	4850 (3455‐6980)	5745 (3483‐7783)	0.862
IgG3, M (Q1‐Q3) mg/L	500 (362‐830)	474 (149‐632)	395 (127‐860)	0.410
IgG4, M (Q1‐Q3) mg/L	12 250 (5568‐15 625)	4725 (2738‐8748)	6020 (2613‐11 450)	0.01^a^
IgE, M (Q1‐Q3) KU/L	473 (55.9‐969.8)	17 6(22.3‐615.5)	145 (61.2‐416.8)	0.001^a^
IgG4‐RD RI	10.06 ± 4.17	3.61 ± 2.44	3.13 ± 1.71	<0.0001^a^
PGA	1.26 ± 0.29	0.54 ± 0.38	0.54 ± 0.40	<0.0001^a^

Note

The normal range of serum IgG is 7‐17 g/L, IgG4 is 0‐1400 mg/L, IgE is 0‐60 KU/L.

Abbreviations: ESR, erythrocyte sedimentation rate; HGB, baseline hemoglobin; hsCRP, high‐sensitivity C‐reactive protein; IgG4‐RD RI, immunoglobulin G4‐related disease responder index; PGA, physician's global assessment; PLT, platelets; WBC, white blood cells.

a Represents that there was statistical significance.

##### Changes of IgG4‐RD RI

The disease activity was evaluated by IgG4‐RD RI and PGA. The unit of PGA score was 0‐10. The IgG4‐RD RI was 10.06 ± 4.17 at baseline, 3.61 ± 2.44 at 12 weeks and 3.13 ± 1.71 at 24 weeks of follow‐up, *P *<* *0.0001 (Table 1, Figure 1). PGA at baseline, week 12 and week 24 was 4.17 ± 0.97, 1.80 ± 1.25 and 1.80 ± 1.31 respectively, *P *<* *0.0001 (Table 1, Figure 1).

### 3.3 Safety assessment

Of all 30 patients enrolled, no severe adverse reactions were found in this study. During follow‐up, 6 (20%) patients had mild side effects, including three with oral ulcers, two with stomach discomfort, and one with mild hair loss. No patients showed abnormality in blood routine tests, liver function or renal function. Of patients with stomach discomfort, omeprazole and life‐style changes helped alleviate this discomfort (Table S1). Oral ulcers and hair loss were alleviated to some extent by applying vitamin B and reducing the dosage of iguratimod to once a day.

### 3.4 Cytokine levels before and after treatment

Plasma cytokines were tested at baseline and 3 months after treatment. After treatment, IFN‐γ decreased from 0.868 ± 0.374 pg/mL to 0.396 ± 0.331 pg/mL, *P *=* *0.025. BAFF was 792.87 ± 98.44 pg/mL, and decreased to 696.64 ± 86.88 pg/mL after treatment, *P *=* *0.027. There was no significant changes in serum TGF‐β1, IL‐1β, IL‐17A and TNF‐α before and after treatment.

### 3.5 Changes of T and B cell subsets before and after treatment

**Figure 2:**
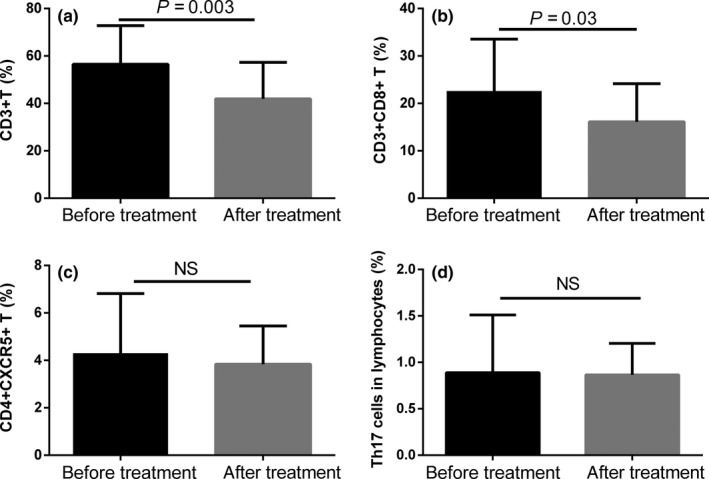
T cells and subpopulations before and after treatment. A‐D represent CD3+ T cells, CD3+ CD8+ T cells, CD4+ CXCR5+ T cells, Th17 cells before and after 3 mo of treatment. NS represented no statistical significance

T cell and B cell subsets were measured before and 3 months after treatment by flow cytometry (Figures S1 and S2). Of T cell subsets, CD3+ T cells were 56.49% ± 16.28% in lymphocytes, which decreased significantly to 41.88% ± 15.4% after 3 months treatment, *P *=* *0.003 (Figure 2). CD3 + CD8 + T cells also decreased from 22.61% ± 10.94% to 16.13% ± 8.02%, *P *=* *0.03 (Figure 2). There was no statistical significance of CD3+ CD4+ T cells, CD4+ CXCR5+ TFH (Figure 2), or the expression of PD‐1 and ICOS on CD4+ CXCR5+ TFH cells before and after treatment. And there was no statistical significance in Th17 cells among healthy controls and IgG4‐RD patients before and after treatment.

**Figure 3:**
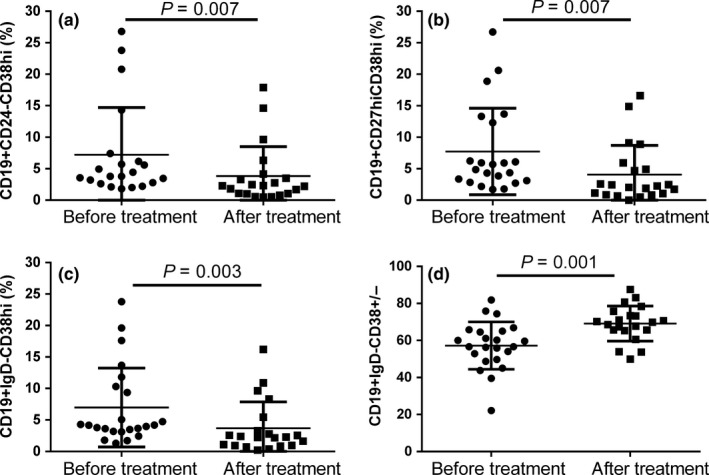
B cells and subpopulations before and after 3 mo of treatment. A‐D represent CD19+ CD24‐CD38hi plasmablast/plasma cells, CD19+ CD27hiCD38hi plasmablasts, CD19+ IgD‐CD38hi plasmablasts and CD19+ IgD‐CD38± naïve B cells, respectively. After treatment, plasmablast/plasma cells decreased significantly, and naïve B cells increased significantly

Of B cell subsets, there was no significance of total CD19+ B cells before (4.52% ± 2.47%) and after (5.11% ± 3.45%) treatment. Plasmablast/plasma cells, expressed as CD19 + CD24‐CD38hi, decreased from 7.21% ± 7.51% to 3.83% ± 4.68% after 3 months treatment, *P *=* *0.007 (Figure 3). CD19 + CD27hiCD38hi plasmablasts also significantly decreased from 7.75% ± 6.87% to 4.07% ± 4.65%, *P *=* *0.007. CD19 + IgD‐CD38hi plasmablasts decreased from 6.98% ± 6.26% to 3.69% ± 4.16%, *P *=* *0.003. Naïve B cells defined as CD19+ IgD+CD38± were increased from 57.23% ± 12.81% to 69.13% ± 9.48%, *P *=* *0.001 (Figure 3). In addition, there was a reduction of CD19+ IgD‐CD38‐CD27+ memory B cells from 10.98% ± 5.36% to 8.14% ± 3.4% after treatment, *P *=* *0.049.

### 3.6 Metabolomics analysis

#### 3.6.1 Global metabolic profiling

**Figure 4:**
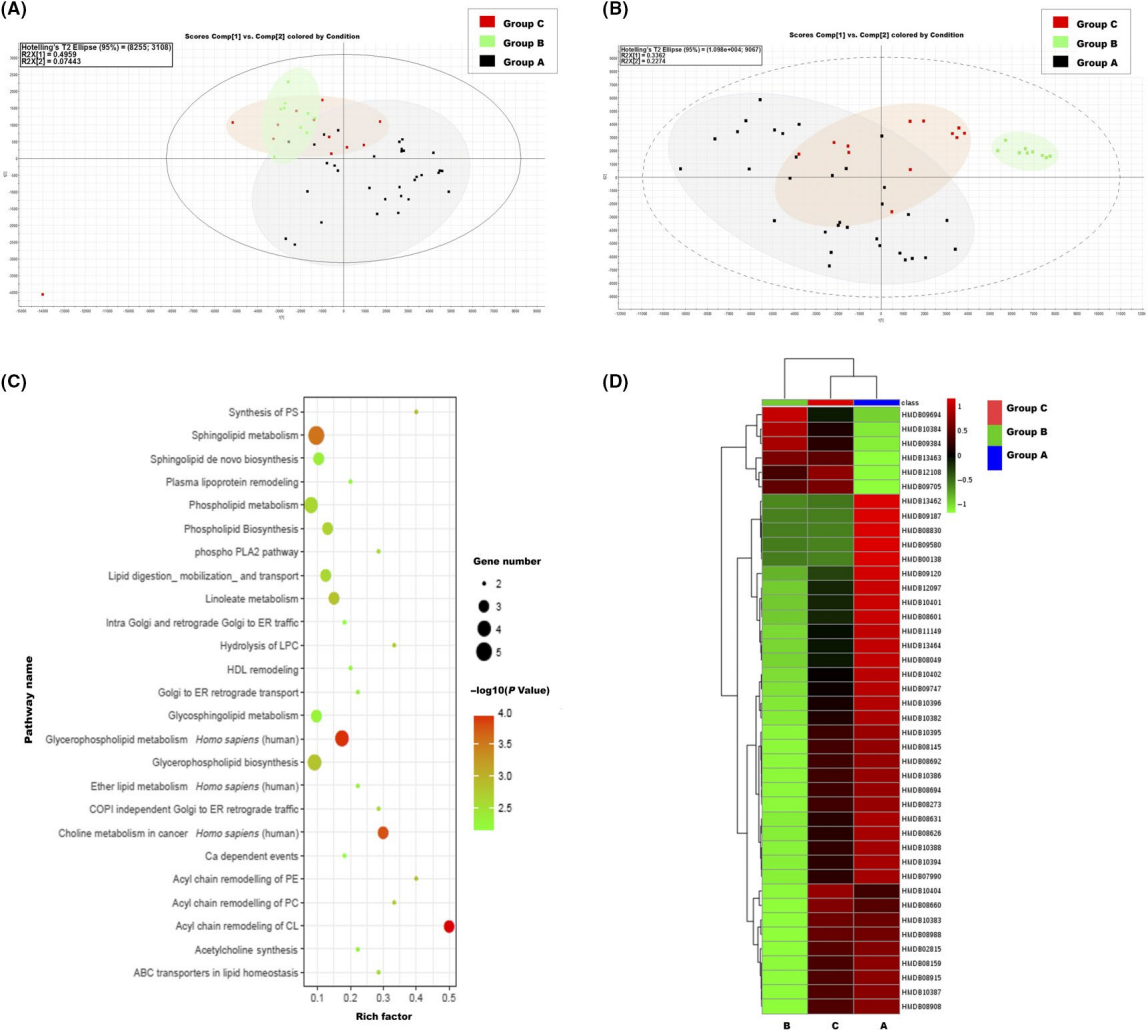
Global metabolic profiling of serum samples. Group A indicates healthy controls, Group B is the initial immunoglobulin G4‐related disease (IgG4‐RD) group while Group C is the iguratimod treatment group. (A) Partial least squares‐discriminant analysis (PLS‐DA) in ESI+ ionization mode; (B) PLS‐DA analysis in ESI‐ ionization mode. (C) Pathway analysis by IMPaLa pathway enrichment. (D) Hierarchical clustering analysis was generated by using MetaboAnalyst. The abundance of biomarkers is shown in the three different groups

In order to characterize the difference in metabolic profiling between the healthy control group (Group A), IgG4‐RD initial group (Group B) and iguratimod treatment IgG4‐RD group (Group C), OPLS‐DA (orthogonal partial least squares‐discriminant analysis) and PLS‐DA (partial least squares‐discriminant analysis) were performed. As shown in Figure S3, a clear separation of Group A and Group B was achieved. To estimate the efficacy of iguratimod treatment, PLS‐DA statistical analysis was conducted among the three groups (Figure 4A,B). The PLS‐DA ESI+ data showed cumulative values of R2(Y) = 55% and Q2 = 43% and PLS‐DA ESI‐ showed R2(Y) = 42% and Q2 = 28%. The results indicated excellent clustering and clear distinction. Group C was located between Groups A and B, but overlapped with Group A in both ionization mode. Distance between samples were measured and clustered by dendrogram clustering; a similar changing trend was observed in the dendrogram clustering results (Figure S4). The results suggested that iguratimod treatment affected systematic metabolic profiles, which restored the metabolic pattern back or near normal levels.

#### 3.6.2 Biomarker identification and metabolic pathway reconstruction

A total of 229 metabolites were significantly changed between Group A and Group B, including 88 in positive ion mode and 141 in negative ion mode. These candidates include amino acid metabolites, carboxylic acids compounds and lipid compounds.

To further explore the metabolic pathways involved in IgG4‐RD, the identified metabolites were subjected to software IMPaLA (Table S2). As shown in Figure 4C, there were multiple metabolic pathways (*P *<* *0.05) constructed with the corresponding metabolites, including glycerophospholipid metabolism, sphingolipid metabolism and choline metabolism. We found phosphatidylcholines (PCs), lyso‐phospatidylcholines (lyso‐PCs), phosphatidylethanolamine, sphingomyelins (SMs) and glycocholic acid were involved in most of these pathways. It suggested that these compounds were significantly associated with IgG4‐RD and might be potential indicators of IgG4‐RD (Table S3). Thus, heatmap analysis was performed to assess the variation in the three groups (Figure 4D). Compared with healthy controls, biomarkers were significantly changed in initial IgG4‐RD patients. After iguratimod treatment, most of these metabolites were reversed to near normal. The result revealed that iguratimod treatment could ameliorate the abnormal state of metabolism induced by IgG4‐RD.

## 4 DISCUSSION

In this study, iguratimod with corticosteroid as bridge treatment for IgG4‐RD with mild symptoms was effective. Patients’ clinical symptoms alleviated, IgG4‐RD RI and PGA decreased, as well as reduction of laboratory parameters including serum IgG, IgA, IgM, IgG4, IgE and ESR. In addition, CD3+ T cells and CD3+ CD8+ T cells, as well as memory B cells and plasmablast/plasma cells decreased after treatment. Further, IFN‐γ and BAFF in plasma decreased after treatment. Of 30 enrolled IgG4‐RD patients with mild symptoms, at week 24, 9 (30%) patients had CR, 17 (56.7%) patients had PR and 4 (13.3%) patients did not respond to iguratimod treatment. By metabolite profiling, metabolites of iguratimod‐treated patients were altered and approached to the status of healthy controls rather than untreated patients. Meanwhile, phospholipids were significantly associated with IgG4‐RD and might be potential indicators of IgG4‐RD. To our knowledge, this study was the first to evaluate the efficacy and safety of iguratimod in the treatment of IgG4‐RD.

Glucocorticoids were considered as the first‐line agent of IgG4‐RD;^17^, ^18^ however by glucocorticoids tapering, patients may suffer disease relapse and long‐term glucocorticoids usage may cause many undesirable adverse drug reactions, especially in older patients. Immunosuppressants combined with glucocorticoids treatment could reduce the relapse rate of IgG4‐RD; however, it was reported immunosuppressants intake alone had limited treatment efficacy.^5^, ^18^, ^19^ For the mild IgG4‐RD patients, in this study, in order to avoid adverse drug reactions by long‐term glucocorticoids, patients were given one dose of diprospan, and then iguratimod orally 25 mg twice a day. From our experience, after diprospan injection, most patients had obvious improvement within 2 weeks; however, a majority of patients could have disease relapse at around 1 month of treatment. So, we designed this clinical trial by using one dose of diprospan combined with iguratimod treatment, with the aim of long‐term disease alleviation.

Iguratimod is a member of the methane sulfonanilide family; although most of the members of this family act as cyclo‐oxygenase 2 (COX 2) inhibitors, iguratimod was considered as a novel immunomodulator based on more and more evidence. Further, iguratimod was also recommended as a disease‐modifying anti‐rheumatic drug by Japan, the Asia‐Pacific League of Associations for Rheumatology Congress and the Chinese guidelines on the treatment of RA. Existing studies revealed that iguratimod reduced the production of cytokines including IFN‐1β, IL‐6, IL‐8 and IL‐17A both in vitro and in vivo.^20^ Further, iguratimod could reduce immunoglobulin production in both mice and humans with RA.^21^ Iguratimod could also attenuate proteinuria and kidney injury in MRL/lpr lupus mice, and improve serum markers such as anti‐dsDNA and serum C3.^12^ In addition, there are some studies published in Chinese which showed iguratimod treatment was effective for primary Sjögren's syndrome and patient's serum Ig was decreased as well. As we know, one of the most prominent characteristics of IgG4‐RD is activation of B cells and plasmablast cells, which excrete large amount of serum IgG and IgG4.^3^, ^6^, ^22^, ^23^ Therefore, we think that iguratimod treatment may be effective in the treatment of IgG4‐RD.

Among our patients, 86.7% responded to iguratimod treatment, including 9 (30%) patients with CR and 17 (56.7%) with PR. Except for three patients suffering oral ulcers, two patients with stomach discomfort, and one patient with mild hair loss, there was no severe adverse drug reaction during treatment. In addition, CD3+ T cells and CD3+ CD8+ T cells were decreased after treatment, whereas there was no significant change in Th17 cells, as well as IL17A secretion in CD4+ T cells, which was not consistent with the findings in RA patients,^24^ indicating a different pathogenesis between IgG4‐RD and RA. In terms of B cells, naïve B cells elevated, while memory and plasmablast/plasma cells decreased after treatment. We, and Wallace et al all revealed that circulating plasmablast/plasma cells could be biomarkers for IgG4‐RD patients,^22^, ^25^ thus the reduction of plasmablast/plasma cells could be considered as an indicator of effective iguratimod treatment.

Except for the improvement of clinical manifestations, patients’ serum levels of IgG, IgG4, IgA, IgM, ESR and IgE were all decreased at treatment, consistent with the study of iguratimod in the treatment of RA.^21^, ^26^ Further, plasma IFN‐γ and BAFF was also decreased after treatment. BAFF is a critical factor for B cell survival and maturation which regulates B cell survival through interaction with their receptor BAFF‐R.^27^, ^28^ Increased BAFF‐induced B cell differentiation into GC B cells within the autoimmune target tissue, the decreased levels of BAFF in plasma after iguratimod treatment may indicate B cells in the pathogenesis and efficacy of iguratimod treatment.^28^, ^29^ From our study, patients with mild disease tended to have higher levels of serum IgG and IgG4 than those without mild disease.^30^ After iguratimod treatment, patients’ serum immunoglobulin reduced as well as disease activity. Twenty percent of patients suffered mild adverse drug reactions, and by adjusting life styles and symptomatic treatment, adverse drug reactions alleviated. Our study indicated that iguratimod treatment was a safety drug in the treatment of IgG4‐RD with mild symptoms, consistent with Xiao et al's^31^ study in healthy adult volunteers.

This is the first study evaluating therapeutic effects and mechanisms of iguratimod on IgG4‐RD through biochemistry and metabolomics. In the present study, we have demonstrated the LC‐MS‐based metabolic profile of initial IgG‐RD patients from iguratimod‐treated patients and healthy controls. Between IgG4‐RD patients and healthy controls, metabolites with statistical significance were mainly involved in glycerophospholipid metabolism, choline metabolism and phospholipid metabolism pathway. Phospholipids were found to be a potential panel of IgG4‐RD. Iguratimod treatment could provide significant effects on IgG4‐RD through restoring multiple pathways and related biomarkers to normal levels, similar to patients treated with glucocorticoids combined with/without immunosuppressants (data not shown). Therefore, iguratimod is a promising drug in the application of IgG4‐RD treatment.

This study had some limitations. First, this was a one‐arm study without a control group. As there was no “golden” or “anchor” immunosuppressant drug for treatment of IgG4‐RD, we did not set controls for this trial. Second, all patients were given one dose of diprospan; we should take into consideration that diprospan might affect the evaluation of iguratimod. Third, patients and doctors were not blinded which may have contributed to some biases.

In conclusion, iguratimod plus corticosteroid as bridge therapy is effective for the treatment of mild IgG4‐RD; it can improve the clinical symptoms of patients, reduce the serum IgG and IgG4 levels, and can also reduce the peripheral CD3+ CD8+ T cells, and especially plasmablasts and memory B cells. Iguratimod is well tolerated in IgG4‐RD patients. Clinical trial registration number: NCT03368274.

## CONFLICT OF INTEREST

The authors disclose no conflicts.

## AUTHOR CONTRIBUTIONS

PZ and YG participated in flow cytometric analysis, cell culturing, ELISA, CBA, metabolic profiling and statistical analysis, and they drafted the manuscript. ZL,WL, JQL carried out flow cytometric analysis. YL, MW, XL, YF, HC, LP, JL, JZ, QS, XZ, MS, XZ, FZ and YZL helped with recruiting patients, study conception and design, and revision of the manuscript. WZ and YZ conceived of the study, participated in its design and coordination, and helped to draft the manuscript. All authors read and approved the final manuscript.
