# Supplementary material for: Efficacy and safety of iguratimod plus corticosteroid as bridge therapy in treating mild IgG4‐related diseases: A prospective clinical trial
Source: Int J Rheum Dis. 2019 Jun 27;22(8):1479–88. doi: 10.1111/1756-185X.13633 (PMC6772123; doi:10.1111/1756-185X.13633)
Supplement: Supplementary file 1 [file APL-22-1479-s001.docx]

**Contents**

**Method details2**

Flowcytometry analysis**2**

Metabolomics analysis **3**

**Supplementary tables5**

**Supplementary figures11**

# Supplementary methods

**Flowcytometry analysis**

T cell subpopulations were stained with phycoerythrin (PE)-anti-CD8, allophycocyanin(APC)-anti-CD4, fluorescein isothiocyanate (FITC)-anti-CD3, peridinin chlorophyllprotein-CY5.5 (Percp-cy5.5)-anti-CXCR5, FITC-anti-CD4, PE-anti-IL17A, APC-anti-IFN-γ, PE-cyanine 7 (Cy7) -anti ICOS, PE-anti-PD1, FITC-anti-CD3, PE-anti-PD1, and APC- anti-IFN-γ antibodies were from BD bioscience, other antibodies of T cell subpopulation staining were from biolegend; B cell subpopulations were stained with PEcy7-anti-CD19, FITC-anti-CD24, APC-anti-CD38, PE-anti-IgD, Percpcy5.5-anti-CD27 (BD bioscience); plasma cells were stained with APC-anti-CD38, PE-anti-CD138 (BD bioscience) and isotype-matched controls. For surface marker staining, after incubation for 30 minutes at 4 °C, the cells were washed and re-suspended in PBS. For intracellular staining, PBMC were incubated with PMA, ionomycin and golgistop (BD Biosciences) for 4-6 hours in 37 °C incubator, 5% CO_2_. After staining of cell surface marker for 30 minutes, cells undergo the process of fixation/permeabilization (BD bioscience) and intracellular staining according to the manufacturer’s instructions. All flowcytometry experiments were performed and analyzed using BD FACS Aria II system (BD Biosciences).

**Metabolomics analysis**

The serum samples were centrifuge at 14,000×g for 10 min to remove precipitates. Then, 100 μL of serum were transferred into 400 μL methanol (pre-chilled to -80 °C) to make a final 80% (v/v) methanol solution. Sample solutions were incubated at -80 °C for 2-4h followed by 14,000×g for 20 min at 4 °C. All supernatants were dried and re-dissolved in 100 μL of 50% (v/v) methanol solution until analysis. A pool of equal volumes of serum metabolites from all the samples were used as the quality control (QC) for UPLC-MS optimizing and normalizing ^[^[^16^](#_ENREF_16)^]^.

For chromatography separation, a Waters ACQUITY UPLC system was used. The separation was carried out on an ACQUITY UPLC^®^BEH C18 column (1.7 μm, 2.1 × 100 mm, Waters Corporation, USA). Both ionization modes were performed with gradient elution using (A) water with 0.1% formic acid and (B) acetonitrile with 0.1% formic acid as the mobile phase. The total analysis time lasted 20 min at the flow rate of 0.4 mL/min: 0–1.5 min, 5%-20% B; 1.5–15 min, 20%–90% B; 15–18 min, 90% –100% B; 18-20 min, held at 5% B for re-equilibration. The sample injection volume was 4 μL.

A XevoG2-XS Q-TOF Mass Spectrometer (Waters Corp., Milford, USA) was connected to the UPLC system. The capillary voltage, sample cone voltage, and the source temperature were 2.5 kV, 25V, 100◦C, respectively. MS data were acquired from m/z 50 to m/z 2000 in the MS^E^ full scan mode with the acquisition rate at 0.2 s/scan. During MS analysis, a leucine-enkephalin calibrant solution (200 ng/mL) was continuously infused into the MS, generating the reference ion ([M-H]^−^=554.262, [M+H]^+^=556.277) to ensure mass accuracy.

The MS raw data were processed using Progenesis QI software (Waters Corp., USA) to perform peak detection and using EZinfo Ver. 3.0 software (Waters Corp., USA) for pattern recognition and PCA analysis. The candidate metabolites, with VIP (Variable Importance for Projection) >1 and *P* (ANOVA) <0.05, were considered to be potential biomarkers. The molecular of interests were searched against the HMDB database (<http://hmdb.ca>). In addition, heatmap and metabolic pathway enrichment was achieved through IMPaLA (<http://impala.molgen.mpg.de/impala/>) and MetaboAnalyst 4.0 software (<http://www.metaboanalyst.ca/>).

# Supplementary tables

**Table S1. Adverse drug reaction of iguratimod after treatment**


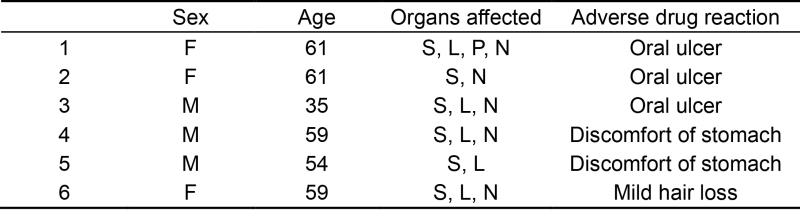


F represented female, and M represented male. S, L, P and N represented submandibular gland, lacrimal gland, parotid gland and nasosinus respectively.

**Table S2. Metabolic pathways constructed by IMPaLa.**

| pathway name | pathway source | num all pathway metabolites | P value | num overlapping metabolites | Rich factor |
| --- | --- | --- | --- | --- | --- |
| Acyl chain remodeling of CL | Reactome | 6 | 2.90E-05 | 3 | 0.5 |
| Glycerophospholipid metabolism | KEGG | 23 | 0.000119 | 4 | 0.173913 |
| Choline metabolism in cancer | KEGG | 10 | 0.000168 | 3 | 0.3 |
| Sphingolipid metabolism | Reactome | 52 | 0.00028 | 5 | 0.096154 |
| Synthesis of PS | Reactome | 5 | 0.00132 | 2 | 0.4 |
| Acyl chain remodelling of PE | Reactome | 5 | 0.00132 | 2 | 0.4 |
| Linoleate metabolism | EHMN | 20 | 0.00148 | 3 | 0.15 |
| Glycerophospholipid biosynthesis | Wikipathways | 44 | 0.00153 | 4 | 0.090909 |
| Acyl chain remodelling of PC | Reactome | 6 | 0.00197 | 2 | 0.333333 |
| Hydrolysis of LPC | Reactome | 6 | 0.00197 | 2 | 0.333333 |
| Phospholipid Biosynthesis | SMPDB | 23 | 0.00224 | 3 | 0.130435 |
| Phospholipid metabolism | Reactome | 50 | 0.00248 | 4 | 0.08 |
| Lipid digestion mobilization and transport | Wikipathways | 24 | 0.00254 | 3 | 0.125 |
| ABC transporters in lipid homeostasis | Reactome | 7 | 0.00273 | 2 | 0.285714 |
| COPI-independent Golgi-to-ER retrograde traffic | Reactome | 7 | 0.00273 | 2 | 0.285714 |
| phospho-PLA2 pathway | Reactome | 7 | 0.00273 | 2 | 0.285714 |
| Sphingolipid de novo biosynthesis | Reactome | 29 | 0.00441 | 3 | 0.103448 |
| Acetylcholine Synthesis | Wikipathways | 9 | 0.00462 | 2 | 0.222222 |
| Ether lipid metabolism | KEGG | 9 | 0.00462 | 2 | 0.222222 |
| Golgi-to-ER retrograde transport | Reactome | 9 | 0.00462 | 2 | 0.222222 |
| pathway name | **pathway source** | **num all pathway metabolites** | **P value** | **num overlapping metabolites** | **Rich factor** |
| Glycosphingolipid metabolism | Reactome | 31 | 0.00534 | 3 | 0.096774 |
| Plasma lipoprotein remodeling | Reactome | 10 | 0.00573 | 2 | 0.2 |
| HDL remodeling | Reactome | 10 | 0.00573 | 2 | 0.2 |
| Intra-Golgi and retrograde Golgi-to-ER traffic | Wikipathways | 11 | 0.00696 | 2 | 0.181818 |
| Ca-dependent events | Reactome | 11 | 0.00696 | 2 | 0.181818 |

**Table S3. Biomarker identification and abundance**

| HMDB ID | Identification | Group B intensity (Average ±SD) ×10^2^ | Group C intensity (Average ±SD) ×10^2^ | Group A intensity (Average ±SD) ×10^2^ | *p*-value（ANOVA) | *p*-value (Group B-Group C) | *p*-value (Group A-Group B) | *p*-value (Group A-Group C) |
| --- | --- | --- | --- | --- | --- | --- | --- | --- |
| HMDB13464 | SM(d18:0/16:1(9Z)) | 156.28±36.95 | 728.08±1117.77 | 1409.72±1532.35 | 0.0002 | NS | 0.0106 | NS |
| HMDB13463 | SM(d18:0/16:1(9Z)(OH)) | 147.38±72.18 | 137.01±166.48 | 39.70±35.49 | 0.0034 | NS | 1.55E-08 | 0.0020 |
| HMDB13462 | SM(d18:0/14:1(9Z)(OH)) | 5.24±3.34 | 14.16±21.10 | 217.16±184.04 | 0.0301 | NS | 0.0008 | 0.0003 |
| HMDB12108 | LysoPC(17:0) | 233.28±125.09 | 260.09±149.09 | 132.86±78.81 | 2.57E-05 | NS | 0.0107 | 0.0005 |
| HMDB12097 | SM(d18:1/14:0) | 13.62±7.27 | 41.55±53.42 | 84.37±59.16 | 4.39E-08 | NS | 0.0006 | 0.0190 |
| HMDB11149 | LysoPC(O-18:0) | 266.26±101.08 | 458.56±170.64 | 669.61±176.01 | 2.46E-12 | 0.0050 | 1.10E-07 | 0.0006 |
| HMDB10404 | LysoPC(22:6(4Z,7Z,10Z,13Z,16Z,19Z)) | 1423.12±443.06 | 2427.37±554.72 | 2180.88±1047.27 | 0.0047 | 0.0002 | 0.0283 | NS |
| HMDB10402 | LysoPC(22:5(4Z,7Z,10Z,13Z,16Z)) | 265.52±89.02 | 362.02±114.44 | 453.00±256.16 | 0.0063 | 0.0303 | 0.0245 | NS |
| HMDB10401 | LysoPC(22:4(7Z,10Z,13Z,16Z)) | 340.68±109.94 | 457.47±105.66 | 639.44±240.86 | 9.36E-08 | 0.0151 | 0.0007 | 0.0088 |
| HMDB10396 | LysoPC(20:4(8Z,11Z,14Z,17Z)) | 5912.74±1659.91 | 11696.38±2471.0 | 15934.44±4450.3 | 0 | 7.29E-06 | 7.47E-08 | 0.0020 |
| HMDB10395 | LysoPC(20:4(5Z,8Z,11Z,14Z)) | 1258.09±352.37 | 2098.95±505.63 | 2405.85±883.75 | 3.17E-07 | 0.0003 | 0.0004 | NS |
| HMDB10394 | LysoPC(20:3(8Z,11Z,14Z)) | 3034.60±528.47 | 5524.26±1293.33 | 6816.22±2557.04 | 6.60E-10 | 2.67E-05 | 5.35E-05 | NS |
| HMDB10388 | LysoPC(18:3(9Z,12Z,15Z)) | 634.93±604.83 | 925.37±708.23 | 1061.33±1973.99 | 0.0113 | NS | NS | NS |
| HMDB10387 | LysoPC(18:3(6Z,9Z,12Z)) | 206.28±71.95 | 352.89±220.93 | 381.55±236.62 | 5.82E-05 | 0.0421 | 0.0152 | NS |
| HMDB10386 | LysoPC(18:2(9Z,12Z)) | 3373.12±894.31 | 6755.70±1205.17 | 7870.28±2927.20 | 1.36E-12 | 9.71E-07 | 4.83E-05 | NS |
| HMDB10384 | LysoPC(18:0) | 5593.97±647.91 | 5008.36±763.90 | 4125.14±1207.75 | 1.15E-14 | 0.0470 | 0.0009 | 0.0130 |
| HMDB10383 | LysoPC(16:1(9Z)) | 1164.41±308.70 | 1825.53±487.33 | 1819.10±944.69 | 0.0031 | 0.0015 | 0.0224 | NS |
| HMDB10382 | LysoPC(16:0) | 201.13±26.00 | 382.81±69.69 | 498.34±88.09 | 0 | 4.91E-07 | 3.49E-12 | 0.0001 |
| HMDB09747 | PE(24:1(15Z)/15:0) | 3570.22±711.70 | 7509.18±3251.45 | 10988.54±5478.8 | 1.16E-09 | 0.0015 | 0.0002 | 0.0249 |
| HMDB ID | **Identification** | **Group B intensity (Average ±SD) ×10^2^** | **Group C intensity (Average ±SD) ×10^2^** | **Group A intensity (Average ±SD) ×10^2^** | ***p*-value（ANOVA)** | ***p*-value (Group B-Group C)** | ***p*-value (Group A-Group B)** | ***p*-value (Group A-Group C)** |
| HMDB09705 | PE(22:6(4Z,7Z,10Z,13Z,16Z,19Z)/22:6(4Z,7Z,10Z,13Z,16Z,19Z)) | 1322.94±190.07 | 1373.79±1119.90 | 635.95±523.15 | 0.0169 | NS | 0.0004 | 0.0030 |
| HMDB09694 | PE(22:6(4Z,7Z,10Z,13Z,16Z,19Z)/20:3(5Z,8Z,11Z)) | 1128.73±275.36 | 813.55±479.04 | 569.56±429.11 | 0.0051 | NS | 0.0002 | NS |
| HMDB09580 | PE(22:4(7Z,10Z,13Z,16Z)/14:0) | 9.16±1.59 | 6.48±4.11 | 203.46±180.52 | 0.0097 | 0.0466 | 0.0016 | 0.0003 |
| HMDB09384 | PE(20:4(5Z,8Z,11Z,14Z)/15:0) | 1828.09±812.20 | 1507.02±832.21 | 920.92±618.02 | 0.0012 | NS | 0.0002 | 0.0087 |
| HMDB09187 | PE(18:4(6Z,9Z,12Z,15Z)/16:0) | 0.86±0.97 | 0.88±2.35 | 95.12±83.70 | 0.0004 | NS | 0.0010 | 0.0002 |
| HMDB09120 | PE(18:3(6Z,9Z,12Z)/15:0) | 58.38±12.70 | 162.25±39.00 | 443.32±166.87 | 0 | 3.08E-07 | 2.63E-08 | 6.46E-07 |
| HMDB08988 | PE(18:0/15:0) | 155.84±51.29 | 380.22±318.50 | 389.77±232.35 | 0.0229 | 0.0305 | 0.0028 | NS |
| HMDB08915 | PE(15:0/24:1(15Z)) | 8454.24±2073.77 | 20450.52±8832.6 | 22986.92±11606 | 3.80E-06 | 0.0006 | 0.0005 | NS |
| HMDB08908 | PE(15:0/22:1(13Z)) | 24511.73±4876.1 | 82761.12±48586.5 | 94526.27±45388.5 | 7.20E-11 | 0.0015 | 2.37E-05 | NS |
| HMDB08830 | PE(14:0/18:3(6Z,9Z,12Z)) | 0.84±0.82 | 0.79±2.39 | 126.51±112.58 | 0.0004 | NS | 0.0011 | 0.0002 |
| HMDB08694 | PC(22:5(7Z,10Z,13Z,16Z,19Z)/18:0) | 626.96±304.06 | 1573.33±1195.11 | 1883.34±1434.23 | 0.0008 | 0.0199 | 0.0071 | NS |
| HMDB08692 | PC(22:5(7Z,10Z,13Z,16Z,19Z)/16:0) | 222.84±80.34 | 449.19±433.24 | 521.68±348.82 | 0.0001 | NS | 0.0109 | NS |
| HMDB08660 | PC(22:5(4Z,7Z,10Z,13Z,16Z)/16:1(9Z)) | 34314.95±5097.9 | 63780.4±229105.5 | 60086.45±36300.8 | 0.0324 | 0.0009 | 0.0244 | NS |
| HMDB08631 | PC(22:4(7Z,10Z,13Z,16Z)/18:2(9Z,12Z)) | 8931.06±3228.93 | 13734.90±7514.07 | 16454.58±11384.69 | 0.0229 | NS | 0.0386 | NS |
| HMDB08626 | PC(22:4(7Z,10Z,13Z,16Z)/16:0) | 1764.97±328.76 | 3200.64±1034.60 | 3985.13±1465.40 | 8.62E-07 | 0.0006 | 5.08E-05 | NS |
| HMDB08601 | PC(22:2(13Z,16Z)/18:4(6Z,9Z,12Z,15Z)) | 924.73±178.16 | 1599.16±677.40 | 2651.48±1375.77 | 2.24E-08 | 0.0061 | 0.0004 | 0.0086 |
| HMDB ID | **Identification** | **Group B intensity (Average ±SD) ×10^2^** | **Group C intensity (Average ±SD) ×10^2^** | **Group A intensity (Average ±SD) ×10^2^** | ***p*-value（ANOVA)** | ***p*-value (Group B-Group C)** | ***p*-value (Group A-Group B)** | ***p*-value (Group A-Group C)** |
| HMDB08273 | PC(20:0/18:4(6Z,9Z,12Z,15Z)) | 13222.75±3312.2 | 32160.80±15768 | 38342.4±23910.1 | 0.0004 | 0.0016 | 0.0020 | NS |
| HMDB08159 | PC(18:2(9Z,12Z)/P-16:0) | 5488.99±1953.97 | 9360.71±2582.81 | 10278.81±5219.9 | 0.0159 | 0.0010 | 0.0073 | NS |
| HMDB08145 | PC(18:2(9Z,12Z)/20:2(11Z,14Z)) | 131.48±30.60 | 375.39±271.85 | 448.37±282.41 | 4.71E-07 | 0.0098 | 0.0011 | NS |
| HMDB08049 | PC(18:0/20:4(8Z,11Z,14Z,17Z)) | 55.80±13.14 | 334.31±486.02 | 685.57±839.99 | 1.03E-06 | NS | 0.0172 | NS |
| HMDB07990 | PC(16:0/22:5(7Z,10Z,13Z,16Z,19Z)) | 10464.04±2789.9 | 19701.58±5513.2 | 24370.77±8807.2 | 7.95E-11 | 0.0002 | 3.85E-05 | NS |
| HMDB02815 | LysoPC(18:1(9Z)) | 9418.22±2239.31 | 19327.61±4466.1 | 20715.20±7537.7 | 1.16E-12 | 6.77E-06 | 6.25E-05 | NS |
| HMDB00138 | Glycocholic acid | 19.05±19.42 | 8.14±7.66 | 557.47±2829.06 | 0.0407 | NS | NS | NS |
| HMDB00086 | Glycerophosphocholine | 39.23±21.52 | 64.72±38.89 | 201.84±133.83 | 2.13E-10 | NS | 0.0006 | 0.0007 |

# Supplementary figures

**Figure S1. Typical pictures of T cell subsets**

**

**

**Figure A to D represented CD3+T cells, CD4+CD8+ T cells, CD4+CXCR5+ Tfh cells and IFN-γ+IL17A+ / IFN-γ-IL17A+ in CD4+ in CD4+ T cells respectively.**

**Figure S2. Typical pictures of T cell subsets**

**

**

**Figure A to D represented typical pictures of CD38hiIgD- cells, CD38-IgD-CD27+cells, CD38hiCD27hi cells and CD24-CD38hi cells in CD19+ B cells before and after treatment.**

**Figure S3. OPLS-DA analysis between group A and group B**

**
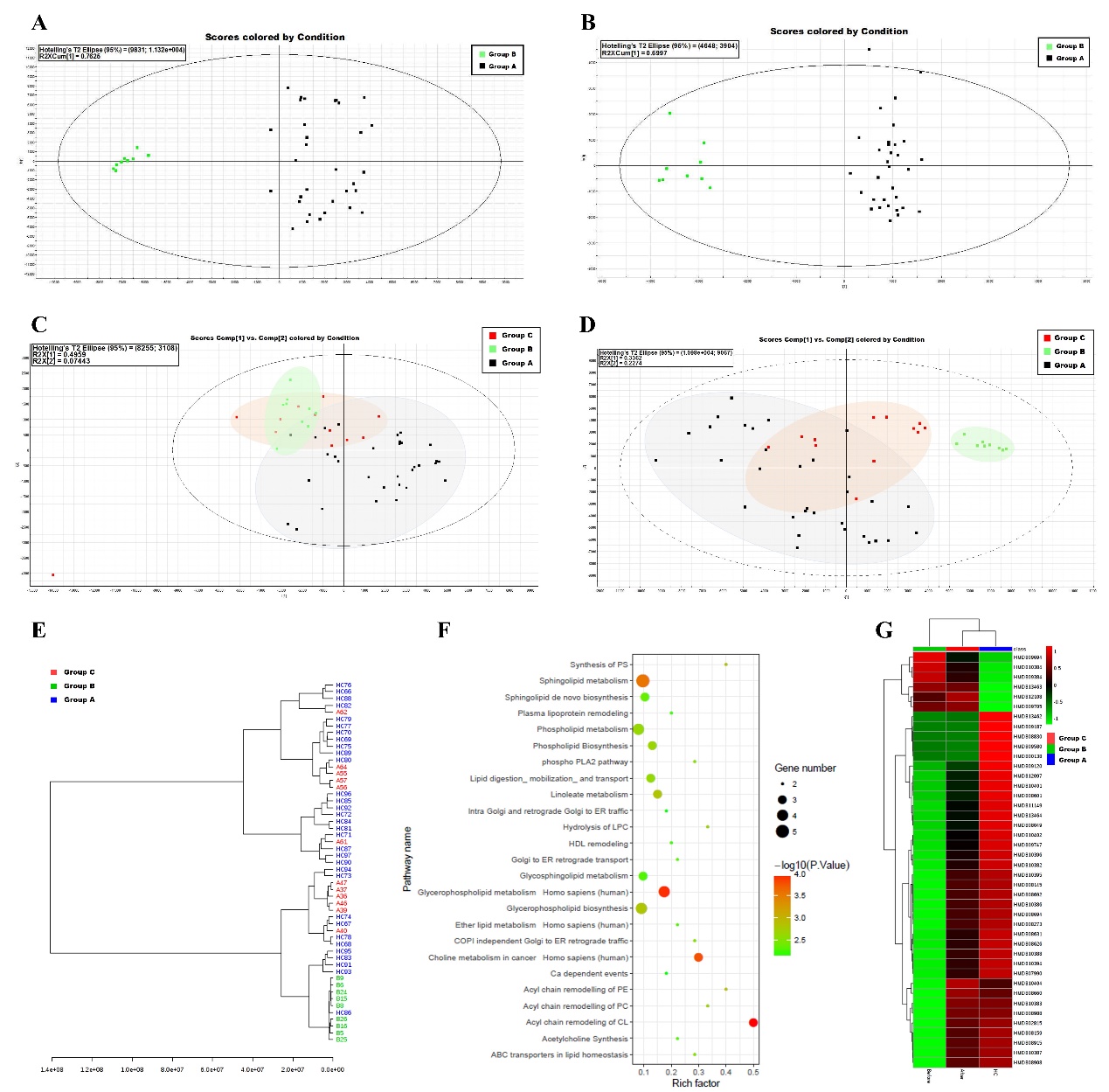
**

Group A indicated healthy control, group B was initial IgG4-RD group: (A) OPLS-DA analysis between group A and group B in ESI+ ionization mode, (B) OPLS-DA analysis between group A and group B in ESI- ionization mode. The OPLS-DA ESI+ data showed cumulative values of R2(Y) = 91% and Q2 = 80% and OPLS-DA ESI- showed R2(Y) = 96% and Q2 = 73%.

**Figure S4. Dendrogram cluster of two groups**


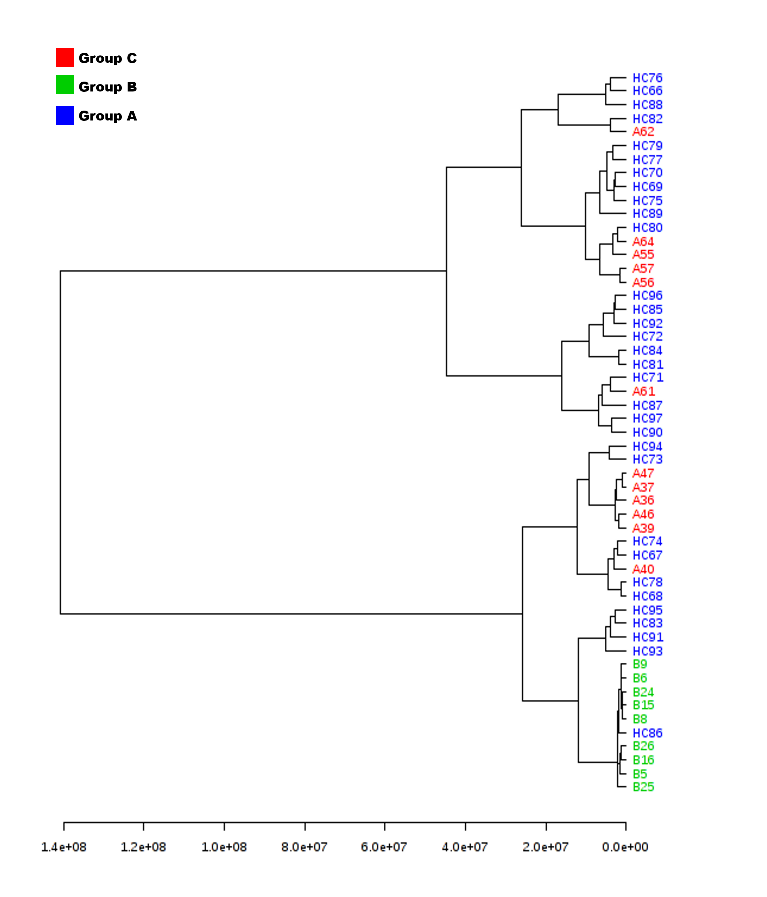


Group A indicated healthy control, group B was initial IgG4-RD group while group C was Iguratimod treatment group. Dendrogram clustering of three groups. Distance between samples were measured and clustered.
